# The Potential Use of Pharmacological Agents to Modulate Orthodontic Tooth Movement (OTM)

**DOI:** 10.3389/fphys.2017.00067

**Published:** 2017-02-08

**Authors:** Thaleia Kouskoura, Christos Katsaros, Stephan von Gunten

**Affiliations:** ^1^Department of Orthodontics and Dentofacial Orthopedics, School of Dental Medicine, University of BernBern, Switzerland; ^2^Institute of Pharmacology, University of BernBern, Switzerland

**Keywords:** orthodontics, orthodontic tooth movement, tooth movement, pharmacology, pharmacological agents

## Abstract

The biological processes that come into play during orthodontic tooth movement (OTM) have been shown to be influenced by a variety of pharmacological agents. The effects of such agents are of particular relevance to the clinician as the rate of tooth movement can be accelerated or reduced as a result. This review aims to provide an overview of recent insights into drug-mediated effects and the potential use of drugs to influence the rate of tooth movement during orthodontic treatment. The limitations of current experimental models and the need for well-designed clinical and pre-clinical studies are also discussed.

## Introduction

During orthodontic treatment, the application of sustained force on teeth sets in motion processes that ultimately lead to alveolar bone remodeling. These biochemical processes involve a multitude of cellular and molecular networks (Ren and Vissink, 2008; Zainal Ariffin et al., 2011; d'Apuzzo et al., 2013; Patil et al., 2013). Pharmacological agents have the potential to interfere with the biochemical processes which govern tooth movement during, and stability after, orthodontic treatment. As a result, the possibility to accelerate/enhance OTM where needed (such as in areas of space closure) and to halt tooth movement where desired (to provide anchorage or to ensure positional tooth stability during the initial retention period) has attracted considerable interest in the field. Already in 1982 Yamasaki et al. showed that local administration of prostaglandins E1 or E2 accelerated experimental tooth movement in monkeys (Macaca fuscata) (Yamasaki et al., 1982).

In the present article we review reported experimental results on drugs that can modulate orthodontic tooth movement (OTM) and discuss the potential of pharmacological strategies aimed at supporting orthodontic interventions.

### Molecular players involved in orthodontic tooth movement (OTM)

A necessary prerequisite for selecting, designing and testing suitable molecules to influence OTM is a detailed knowledge of the role of the different cellular and molecular components driving the biological process of OTM.

To achieve OTM, mechanical forces are applied on teeth. This initially causes fluid movement within the periodontal ligament (PDL) space and distortion of the PDL components (cells, extracellular matrix, and nerve terminals), setting into motion the process of release of a multitude of molecules (neurotransmitters, cytokines, growth factors, arachidonic acid metabolites etc.) which initiate alveolar bone remodeling (Krishnan and Davidovitch, 2006).

Orthodontic load strains nerve endings present in the PDL. These release in response a number of neuropeptides (substance P, vasoactive intestinal polypeptide, and calcitonin gene-related peptide-CGRP), which act on capillaries and cause the adhesion and migration of blood leukocytes into the area of compression (Krishnan and Davidovitch, 2006). These very active cells release signaling proteins (cytokines, growth factors) with a direct effect on cells of the periodontium. In addition, local hypoxia (unavoidably caused in areas of compression by occlusion of the PDL vessels) activates hypoxia-inducible transcription factor (HIF)-1α in endothelial cells and osteoblasts (Dandajena et al., 2012). This leads to expression of downstream genes including VEGF (vascular endothelial growth factor) and receptor activator of NF-kB ligand (RANKL), which mediate the recruitment of peripheral blood mononuclear cells/osteoclast lineage cells from PDL capillaries and their conversion/activation into osteoclasts, respectively.

Furthermore, when cells in the periodontium (fibroblasts, osteoblasts, endothelial and bone lining cells) are subjected to mechanical stress they express cytokines, growth factors and cytokine receptors. Osteoblasts express IL-1b, IL-6, IL-11, TNFa and their receptors in response to compressive stress. IL-b shows an autocrine effect and enhances the phenomenon (Koyama et al., 2008) plus induces osteoblasts to promote osteoclast activity (through induction of RANKL expression). IL-6 is involved in osteoclast recruitment and differentiation. TNFa directly stimulates the differentiation of osteoclast precursors to osteoclasts in the presence of M-CFS (which is a glycoprotein produced by fibroblasts and endothelial cells in response to growth factors and cytokines, such as PDGF, FGF, IL-1, and IL-6). IL-11 enhances the expression of RANKL, a key molecule in osteoclast precursor differentiation, in osteoblasts. In areas of tension, growth factors (e.g., TGF-β) and cytokines (e.g., OPG) produced by PDL cells can induce apoptosis of osteoclasts (Kobayashi et al., 2000) and tip the balance toward bone formation.

One of the immediate responses of the PDL at sites of compression is also the rise in the level of matrix metalloproteinases (MMPs) which are produced by activated fibroblasts.

MMPs either degrade collagen fibers (MMP-1 and MMP-8) or eliminate the degraded collagen (MMP-9 and MMP-2) to allow tooth movement (Lekic and McCulloch, 1996; Cantarella et al., 2006). The degradation of collagen is thought to enhance osteoclast activation, osteoclast migration and adhesion to bone.

Another very important class of molecules expressed by PDL cells under force application (compression or tension) are the chemokines. These are chemotactic cytokines that have been recognized as playing key roles in inflammatory processes but only recently the role of CC inflammatory chemokines in mechanically-induced bone remodeling is starting to become clearer (Andrade et al., 2009; Taddei et al., 2012; Lee et al., 2015). In general, chemokines mediate chemotaxis of leukocytes and bring about cellular differentiation. In the PDL, interaction between CCL2 (chemokine ligand 2) and CCR2 (chemokine receptor 2) have been found to mediate osteoclast precursor attraction to the sites of orthodontic force application and to subsequently induce osteoclast terminal differentiation possibly through their action on RANK and RANKL expression (Taddei et al., 2012). Another chemokine ligand expressed in the PDL under mechanical loading, CCL3, exerts its effects by interacting with chemokine receptors 1 and 5 (CCR1 and CCR5) present on the surface of osteoclasts and osteoblasts. The effects of chemokines seem to be of different nature depending on the receptor to which they bind, as the CCL3-CCR1 interaction leads to the induction of bone resorption by osteoclast recruitment, differentiation/activation (Taddei et al., 2013), while the interaction of chemokines with CCR5 inhibits bone resorption by increasing the production of signals (such as IL-10 and OPG) inhibitory to osteoclast activation (Andrade et al., 2009).

Prostaglandins and leukotrienes are additional players in the process of tooth remodeling. These molecules are produced by enzymatic processing of arachidonic acid, a component derived from phospholipids of cell tissue membranes. They are locally produced hormones with low serum concentration (Smith, 1989). Prostaglandin production can be induced by mechanical stress or by growth factors, hormones and cytokines (such as IL-1, IL-6, and TNF-a) present at the sites of tooth movement. Prostaglandins mediate events such as vasodilation, inflammatory cell recruitment and also act directly on PDL cells.

Prostaglandin E2 (PGE2) is the most widely researched PG with respect to OTM. PGE2 is produced mainly by PDL fibroblasts and osteoblasts (Kanzaki et al., 2002) by the action of the inducible enzyme COX-2 (cyclooxygenase 2), and subsequently by a specific synthase enzyme (PGE synthase). The newly formed PGE2 has different effects depending on the type of transmembrane receptor to which it binds. PGE2 can drive RANKL expression in osteoblasts (by binding to the EP2 or EP4 receptors), which subsequently leads to osteoclast activation (Mayahara et al., 2012), or drive bone mineralization by osteoblasts when binding to the EP1 receptor (Fujieda et al., 1999). In addition, PGE2 has been shown to aid osteoclast formation (Collins and Chambers, 1992) or lead to transient osteoclast inhibition when added to osteoclasts *in vitro* (Fuller and Chambers, 1989).

Leukotrienes are also produced through the enzymatic metabolism of arachidonic acid by 5-lipooxygenase in many cells including osteoclasts or leucocytes chemoattracted to areas of inflammation. The two leukotrienes shown to be involved in tooth movement are LTB4 (leukotriene B4) and LTD4 (a cysteinyl leukotriene), (Moura et al., 2014). Both leukotrienes were found to significantly boost the recruitment and terminal differentiation/activation of osteoclasts through their effect on cytokine synthesis and in the presence of RANKL.

The induction of osteoclasts is also regulated at various levels by inhibitor molecules, such as OPG (whose expression is up-regulated in cells of the periodontium including osteoblasts under tensile stress possibly through the COX pathway of PG synthesis), IL-1RA (a receptor antagonist cytokine which controls the effects of IL-1), IL-12, and IL-10 (which inhibits the RANK osteoclast signaling pathway and other osteoclast stimulating processes) under low stress conditions (Park-Min et al., 2009). In addition, regulatory T cells by direct contact to osteoclasts and through secretion of cytokines such as IL-10 and TGF-β also play a key role in suppressing osteoclastic activity (Zaiss et al., 2007). Balanced osteoclast activity is necessary to prevent uncontrollable osteolysis and control bone metabolism during OTM.

**Figure 1:**
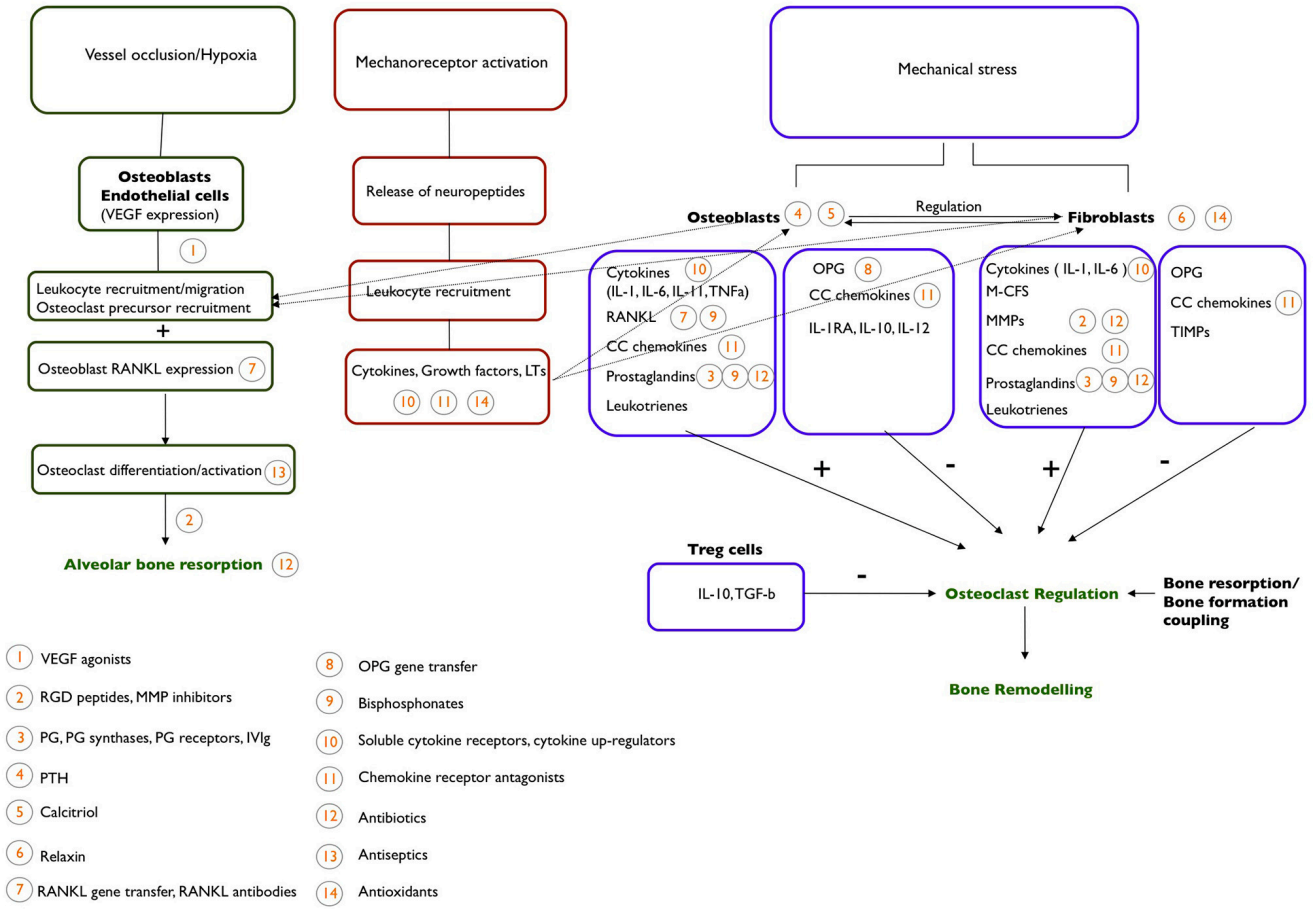
**An outline of the cellular and molecular mechanism behind the process of OTM**. Potential pharmacological agents that could be used to affect OTM and their site of action are indicated.

An outline of the main cellular and molecular components of OTM is shown in Figure 1.

### The search for pharmacological agents to control orthodontic tooth movement

In the last decades an increasing number of pharmacological agents have been explored aiming at the identification of suitable pharmacological means of accelerating or inhibiting OTM. Experimental evidence is mainly based on *in vitro* and animal studies, and a limited number of case-control clinical studies. In the next sections, current knowledge on pharmacological agents that may accelerate or decelerate tooth movement is discussed (Table 1).

**Table 1:** **Agents with proposed potential of accelerating or decelerating orthodontic tooth movement (OTM)**.

**Acceleration of OTM**	**Deceleration of OTM**
Arachidonic acid metabolites	N-acetylcysteine
Cytokines	Bisphosphonates (Clodronate, Aledronate)
1,25-dihydroxycholecalciferol	Chemically modified tetracyclines (CMTs)
RANKL	CetylPyridinium Chloride (CPC)
Parathyroid hormone	Integrin inhibitors
Relaxin	Osteoprotegerin

*Compiled from recent original work and reviews, as discussed in this article. RANKL, receptor activator of NF-kB ligand*.

### Pharmacological acceleration of OTM

#### Arachidonic acid metabolites

Among the arachidonic acid metabolites, PGE2 is by far the most widely tested substance in terms of its capacity to modify OTM. Evidence, mainly derived from animal studies, points toward a positive effect of PGE2 with respect to enhancing bone resorption and accelerating tooth movement (Yamasaki et al., 1982, 1984; Leiker et al., 1995; Kale et al., 2004). The few available clinical studies are of low quality and involve repeated injections of PGE2 and follow-up times of a maximum of 60 days (Camacho and Velásquez Cujar, 2014). The mode of application of PGE2 is a major limitation as it involves repeated injection (due its short half-life) in combination with an anaesthetic solution to alleviate the hyperalgesia caused by injection of PGE2. Potential adverse effects (e.g., root resorption) linked to long-term administration of PGE2, as required in the context of orthodontic treatment, are possible given its mode of action but have not been evaluated so far.

Specific synthases are involved in the pathway of the synthesis of each type of prostaglandins (e.g., PGE and PGD synthases) and many of them have been cloned and could provide drug targets for the regulation of the synthesis of specific prostaglandins, such as PGE2 in the case of OTM (Forsberg et al., 2000). In addition, it is possible that other PGs such as PGI2 may be involved in bone resorption providing further targets for drugs (Wang et al., 1999). Another obvious group of drug targets are the identified receptors of specific prostaglandins (such as the receptors EP1, EP2, or EP4 of prostaglandin PGE2) and the design of selective agonists can provide pharmacological methods of modifying OTM through these receptors.

Intravenous immunoglobulin (IVIg) preparations are polyspecific and polyclonal immunoglobulin therapeutic preparations used as a replacement therapy in immunodeficient patients (Grader-Beck et al., 2011; Schneider et al., 2015). These IVIg preparations were shown to induce COX-2 mediated PGE2 synthesis and cytokine production (Trinath et al., 2013; von Gunten et al., 2013; Djoumerska-Alexieva et al., 2015). It is possible that local administration of these IVIg preparations could be used to modulate bone modeling through PEG2 induction and bypass some of the limitations of PEG2 injections.

#### Hormones (parathyroid hormone, 1,25-dihydroxycholecalciferol, relaxin)

Parathyroid hormone (PTH) exerts its effects directly on osteoblasts and indirectly on osteoclasts through binding to the PTH type 1 receptor on osteoblasts, leading to expression of insulin-like growth factor-1 (IGF-1), which promotes osteoblastogenesis and osteoblast survival, and of RANKL, which promotes osteoclast activation. PTH is also likely interacting with bone lining cells promoting early osteogenesis (Dobnig and Turner, 1995; Esbrit and Alcaraz, 2013)

Recombinant PTH is clinically used for the treatment of osteoporosis and has been shown to mediate bone anabolic or catabolic effects depending on the circumstances of administration (Esbrit and Alcaraz, 2013). Intermittent exposure to PTH seems to increase bone formation, while continuous and long-term exposure (longer than 1–2 years) enhances bone resorption. Studies on the effects of PTH on tooth movement point toward a role of intermittent treatment with PTH in facilitating bone remodeling/turnover and therefore accelerating tooth movement. This is possibly occurring by PTH enhancing both osteoblast and subsequently also osteoclast activity (Li et al., 2013). To date there no long-term studies employing local PTH delivery that discriminate between effects of PTH on tooth movement when different protocols are used (short-term vs. long-term administration and intermittent or continuous mode of administration). Some short-term animal studies indicate that a system of slow, continuous release of PTH in areas of compression is able to accelerate OTM (Soma et al., 1999, 2000).

1,25-dihydroxycholecalciferol or calcitriol is the most active metabolite of vitamin D and has predominantly anabolic, but also catabolic effects on bone. Furthermore, vitamin D also modulates the transcription of genes in immune cells (von Gunten et al., 2013). Calcitriol seems to enhance bone remodeling in a similar way to PTH by enhancing osteoblastic proliferation and function (Reichel et al., 1989), while both PTH and 1,25-dihydroxycholecalciferol have been shown to stimulate PG production in osteoblasts further implicating them in the process of OTM (Pilbeam et al., 1989; Klein-Nulend et al., 1991). Some evidence from animal experiments exists on the effect of local application of calcitriol on tooth movement (Takano-Yamamoto et al., 1992; Kale et al., 2004; Kawakami and Takano-Yamamoto, 2004). During the short experimental time of the available studies there is some evidence that calcitriol enhances the processes of alveolar bone resorption and formation (bone remodeling) leading to acceleration of tooth movement during force application (Takano-Yamamoto et al., 1992; Kale et al., 2004), and also enhances bone formation and remodeling after OTM (Kawakami and Takano-Yamamoto, 2004). A clinical study testing the effects of systemic application of calcitriol indicated that it may produce some enhancement in tooth movement (Blanco et al., 2001).

Relaxin is a peptide hormone with strong effects on collagen turn-over. *In vitro* studies have suggested that relaxin may have a direct effect on the PDL by means of decreasing the expression and release of collagen type I, increasing expression of certain MMPs and decreasing the expression of inhibitors of metalloproteinases (TIMPs), (Henneman et al., 2008; Takano et al., 2008, 2009) in PDL cells. Despite increased interest in the potential of relaxin to modulate OTM, effects on tooth movement and tooth stabilization could to date not be confirmed, neither clinically (McGorray et al., 2012) nor in animal experiments (Stewart et al., 2005; Madan et al., 2007).

### Pharmacological deceleration of OTM

#### OPG and RANKL

The potential use of osteoprotegerin (OPG) to inhibit tooth movement and enhance stability, stems from the known physiological role that OPG plays within the PDL in regulating the bone resorbing activity of osteoclasts. OPG is produced by osteoblasts and is a decoy receptor for RANKL which prevents the interaction of RANKL present on osteoblasts' surface with its receptor RANK on osteoclasts. In the absence of RANKL-RANK interactions, the activation, terminal differentiation and survival of osteoclasts are negatively affected. Changes in the ratio of RANKL/OPG in the PDL can fine-tune alveolar bone resorption. A number of groups attempted to influence tooth movement in animal models by locally altering the concentration of either OPG or RANKL aiming to enhance or decrease the resorptive action of osteoclasts (Kanzaki et al., 2004, 2006; Dunn et al., 2007; Zhao et al., 2012). A local gene transfer method to increase the expression of either OPG or RANKL in periodontal tissues was used in some studies, in which acceleration of tooth movement by gene transfer of RANKL and inhibition by gene transfer of OPG were observed. For the short experimental time periods used, gene transfer is reported to have caused no alterations in bone metabolism in bones distant from the site of injection (Zhao et al., 2012).

Of note is that Denosumab, a humanized monoclonal antibody against RANKL, has recently been added to the list of pharmacological agents used to combat osteoporosis but has not been evaluated with respect to OTM (Bekker et al., 2004; Boyce and Xing, 2008).

#### Bisphosphonates

Bisphosphonates exert a strong inhibitory effect on bone resorption and are successfully used for the treatment of osteoporosis. With respect to the possible effects of different bisphosphonates on tooth movement, *in vitro* studies have shown that both aledronate (Kim et al., 2012) and clodronate (Liu et al., 2006) inhibit the stress-induced up-regulation of key components crucial for mediating tooth movement. Clodronate was shown to reproducibly inhibit the stress-induced expression of COX-2, PGE2, and RANKL in cultured human PDL-derived cells in a concentration-dependent manner (Liu et al., 2006). This action of clodronate is likely to indirectly prevent osteoclast formation and allow osteoclast apoptosis. Kim et al. used chitosan scaffolds loaded with aledronate to investigate possible effects of this bisphosphonate on osteoblasts and on RANKL-induced differentiation of osteoclasts (Kim et al., 2012). They observed concentration-dependent positive effects of aledronate on the proliferation, differentiation and activity of osteoblasts, and a potent inhibitory effect on osteoclast differentiation. An interesting aspect of this study was that chitosan aledronate releasing scaffolds seem to ensure a sustained release of aledronate for a period of 4 weeks, suggesting that this system might provide a feasible delivery vehicle for long-term treatment.

#### Cytokine and chemokine receptor antagonists

Cytokines are released following mechanical stimulation of cells in the PDL. Their effects on initiating tooth movement are mediated through binding to cytokine receptors on target cells. Decreasing the amount of the unbound cytokines could be a possible strategy of reducing tooth movement. This approach was taken in two studies examining the effects of systemic application of soluble cytokine receptors on tooth movement and root resorption in rodents (Zhang et al., 2003; Jäger et al., 2005). The authors concluded that the administration of soluble receptors to IL-1 and TNF-a, or their combination, led to reduction in OTM in all receptor-treated groups by approximately 50% (Jäger et al., 2005). Furthermore, the number and activity of osteoclasts and odontoclasts were reduced.

Another potential approach is to increase the production of osteoclast inhibitory cytokines such as IL-10 and TGF-β. A potential compound with its origins in the chinese medicine is triptolide, which upregulates the production of these cytokines by regulatory T cells (Xu et al., 2016). A water soluble compound of triptolide, minnelide, has recently become available and is currently used in clinical trials to combat pancreatic cancer.

A potential pharmacological approach to decrease bone resorption and tooth movement is through the use of specific chemokine receptor antagonists, such as Met-RANTES, a modified methionylated CCL5 molecule, which binds to both CCR1 and CCR5 receptors and blocks the physiological signaling pathway that leads to bone resorption (Proudfoot et al., 1996; Taddei et al., 2012). Such an intervention could be used to enhance anchorage by preventing the movement of teeth under mechanical loading.

#### Antibiotics/antiseptics

Chemically modified tetracyclines (CMTs) are derivatives of the tetracycline groups of antibiotics that lack antimicrobial activity and the adverse effects associated with the conventional tetracyclines. Their ability to inhibit MMPs and pro-inflammatory cytokines and their apoptotic effects on osteoclasts initially rendered them attractive therapeutic agents for the management of chronic periodontitis. The CMTs have been shown to modify the COX-2 enzyme leading to inhibition of PGE2 production (Patel et al., 1999) and represent also potent inhibitors of MMPs (Marcial et al., 2012). These properties make CMTs a potentially useful pharmacological agent to inhibit tooth movement in order to control anchorage or enhance tooth stability after orthodontic treatment. Initial animal studies (Bildt et al., 2007) showed that oral administration of CMT-3 (now known as COL-3) reduced the rate of tooth movement in rats in a concentration-dependent manner. The exact mechanism of action remains to be elucidated.

Another antibacterial agent, enoxacin, offers an attractive approach for controlling the resorptive activity of osteoclasts. For the resorption of bone mineral, the presence of proton pumps in the ruffled borders of osteoclasts is crucial. To this end, osteoclasts have the unique ability to produce increasing amounts of vacuolar (V) ATPases (proton pumps) upon their activation. These V-ATPases are transported through interaction with microfilaments within the cytoplasm to the plasma membrane- a unique ability limited to clast cells, thus making the targeting of this process a highly specific means for modulation of tooth movement. Enoxacin was first identified by crystal structure-based virtual screening as one of the potential molecules which could block the binding site of V-ATPase to actin (Ostrov et al., 2009). Such interference would in principle prevent transportation of the proton pumps to the ruffled border of the osteoclast. *In vitro* studies confirmed the predicted effect (Ostrov et al., 2009) and animal studies showed that a modified version of enoxasin (bis-enoxacin) with enhanced binding to bone selectively inhibited osteoclasts resulting in inhibition of tooth movement in rats after 28 days (Toro et al., 2013).

There is also some evidence from *in vitro* experiments that the well-known antiseptic Cetyl Pyridinium Chloride (CPC) inhibits RANKL-induced osteoclast formation from bone marrow-derived macrophages possibly by supressing a key event in the RANKL induced intracellular signaling pathway or by interfering with M-CFS signaling (Zheng et al., 2013). To our knowledge potential inhibitory effects of CPC on OTM have not been tested to date. Its already approved use in dental medicine due to its antiseptic properties renders CPC an attractive agent to test for potential beneficial effects on tooth stability after orthodontic treatment.

#### Antioxidants

The theoretical possibility that antioxidants may interfere with tooth movement derives from observations that the cellular concentration of reactive oxygen species (ROS) increases under conditions of hypoxia and mechanical stress in PDL fibroblasts and that hypoxia has been shown to lead to the expression of key genes involved in the recruitment and activation of osteoclasts (Janssen-Heininger et al., 2000; Dandajena et al., 2012). Altered ROS levels also influence the characteristics of immune cells (Wehrli et al., 2014).

The effects of antioxidants (either resveratrol or N-acetylcysteine) were tested in a split mouth design in rats (Chae et al., 2011). The authors reported a significant decrease in tooth movement compared to the control sites following local injection of N-acetylcysteine but not resveratrol.

#### Integrin inhibitors

Integrins are a group of transmembrane glycoproteins, which form heterodimeric units on the membrane of PDL clast cells, such as osteoclasts and odontoclasts (Talic et al., 2004). One of their functions is the adhesion of the differentiated clast cells onto specific proteins on bone (e.g., osteopontin) and dentine enabling them to become polarized and start the bone resorption process, but members of the group mediate other functions such as clast cell migration, cell differentiation and survival.

Integrin molecules adhere to proteins containing RGD (standing for the amino acids arginine, glycine and aspartate) epitopes. Such proteins include osteopontin and bone sialoprotein (Miyauchi et al., 1991). This property has been exploited and a number of studies have used RGD-containing peptides as antagonists to prevent integrin function. With respect to tooth movement, based on results from animal models it appears that there is the potential for RGD peptides to inhibit tooth movement (Dolce et al., 2003) and also root resorption (Talic et al., 2006). There is, however, indication from the experimental results that a more detailed knowledge of the function of the different integrin molecules is crucial, as generalized inhibition of integrin function at PDL sites may have a multitude of undesirable effects.

Another potential means of exploring the role of RGD peptides and inhibiting tooth movement is through the use of MMP inhibitor molecules to reduce the production of RGD peptides by MMPs in areas of compression (Holliday et al., 2003). This animal study used a general (rather than specific) MMP inhibitor, Ilomastat, locally delivered and demonstrated some reduction in bone resorptive osteoclast activity.

### Limitations of the available studies

Tooth movement is a complex process controlled by the nature of the mechanical stimuli, by a multitude of signaling pathways and influenced by the individual's genetic make-up (Zainal Ariffin et al., 2011). Naturally, the huge majority of available *in vivo* experimental evidence derives from animal studies. These have major limitations which include: (1) the inherently different biology in animals which prevents complete inference of the effects and the side effects of the pharmacological agents in humans, (2) the inability to calculate from animal experiments suitable dosages for clinical testing, as systemic application of the drug is often used in these models and may not result in effective or comparable (species difference) drug concentrations at the site of orthodontic intervention, (3) the generally small sample sizes used and different ages of animals used, which makes reliable conclusions even in the animal studies impossible to be reached, (4) the lack of longer-term animal studies, which would enable examination of the effects of an agents on tooth movement over a time period and also allow observation of side-effects.

When clinical human studies are conducted, there are obvious problems related to ethical and practical issues: (1) recruitment of sufficient patient numbers, (2) evaluation of the effect of individual variation, (3) need for initial dose-response studies including measurements to assess the levels of the therapeutic agent at the sites of interest and measurements of the tissue-level outcomes.

The huge majority of the above mentioned studies have employed a systemic administration or local injection of the pharmacological agents. There is multitude of problems associated with these approaches. A systemic administration does not ensure a constant delivery of an ideal dose of the agent in the PDL. As it is not clear how circulating values correspond to the gingival dose and how they fluctuate in time, mainly due to degradation of the agent, in many cases a dose tested in the experimental set-up could have been insufficient to obtain the desired biologic effect. A more important problem is the potential of systemic administration to provoke undesirable systemic effects, especially when pharmacological agents lacking specificity are used. The mere evaluation of side effects is doubtful in the current experimental protocols as either the test periods are too short or the experimental protocol has not included specific methods to evaluate such effects.

Local injection of a pharmacological agent or local gene transfer also come with significant problems. For an intervention to be clinically useful, it must be characterized by practicality in its application (in terms of cost and time) and minimal discomfort for the patient. Daily or even frequent invasive procedures are a major prohibiting factors for clinical application. In addition, during tooth movement different biological processes take place at distinct sites of the PDL. Bone resorption and bone formation occur simultaneously at different areas of the PDL (areas of compression. vs. areas of tension). The proteoglycan component of the extracellular matrix of the PDL, a hydrated gel, allows the diffusion of free small molecules (such as drugs, hormones) within the pdl and presents a big challenge to the effort of achieving targeted therapeutic interventions at specific sites (compressions vs. tension). Most of the drugs used can and will influence both processes and can furthermore influence other physiological cellular functions. The use of integrin inhibitors is a good example of that.

## Future directions

### The need for the design of specific pharmacological agents

Targeting key processes in the bone remodeling mechanism without other undesirable local side effects precludes a detailed knowledge of the cellular events involved. The selection of appropriate targets for the drugs and the design of novel drugs or suitable analogs of naturally occurring molecules with high specificity, is key for clinically successful strategies. The pharmacological agents also need to demonstrate high potency and efficacy in order to achieve clinically significant differences.

The cost implications of the process of designing, testing and eventually obtaining approval for the clinical application of a potential therapeutic agents need to be carefully considered and kept to the minimum to decrease the eventual cost to the patient. A very useful approach as demonstrated by the processes that led to the identification of enoxacin, is to screen already known and approved molecules for clinical applications.

### Development of suitable vehicles of drug delivery and mode of administration

Suitable drug delivery materials need to be developed to provide the appropriate mode of release of pharmacological agents in their active form, that is, at the desired rate and amount for a long period of time (reflecting the duration of orthodontic treatment or retention time). Sustained and low grade prostagladin release through a suitable delivery system could, for example, be used to induce and sustain further endogenous PG production (through a known amplifying mechanism). This can be used to prolong the effects of short periods of stress in OTM.

It is also important that the drug carriers do not have a tendency to spread into a larger area from the application site or that are induced only at areas of bone remodeling (such as matrix-bound inducible molecules). An ideal vehicle for drug delivery must be non-toxic/biocompatible (Démoulins et al., 2013), and be biodegradable (so that no second procedure for its removal is needed) with a suitable half-life and ideally allowing controlled release and alternating delivery of various pharmacological agents, when desired. A convenient method of application (minimal surgical intervention) and reasonable cost are also required to make its clinical application attractive. Materials that have so far been experimentally used for the purpose of delivering drugs to influence tooth movement include the polymer ELVAX (a non-biodegradable polymer) and methylcellulose (in injectable, gel formulation). However, their use was only tested during experimental periods of short duration.

## Author contributions

All authors contributed to conception, data acquisition, drafted manuscript and critically revised the manuscript. All authors gave final approval and agree to be accountable for all aspects of the work.

## Funding

Research by SvG is supported by the Swiss National Science Foundation (SNSF) and the Swiss Cancer League.

### Conflict of interest statement

The authors declare that the research was conducted in the absence of any commercial or financial relationships that could be construed as a potential conflict of interest.
